# The effect of the enhanced recovery programme on long-term survival following liver resection for colorectal liver metastases

**DOI:** 10.1007/s00423-023-02968-4

**Published:** 2023-06-19

**Authors:** Joel Lambert, Thomas Mair, Kalaiyarasi Arujunan, Abdulwarith Shugaba, Harmony Uwadiae, Anne Livesey, Rami Ahmad, Georgios Sgourakis, Christopher Gaffney, Daren Subar

**Affiliations:** 1 BRIDGES Research Group, Department of General and Hepatopancreatobiliary Surgery, East Lancashire Teaching Hospitals NHS Trust, Blackburn, BB2 3HH UK; 2 Lancaster Medical School, Health Innovation One, Sir John Fisher Drive, Lancaster University, Lancaster, LA1 4AT UK

**Keywords:** Enhanced recovery programme, ERP, Colorectal cancer

## Abstract

**Background:**

Enhanced recovery programmes are associated with improved short-term outcomes following liver surgery. The impact of enhanced recovery programmes on medium- and long-term outcomes is incompletely understood. This study aimed to assess the impact of an enhanced recovery programme on long-term survival in patients undergoing surgery for colorectal liver metastases.

**Methods:**

At a tertiary hepatobiliary centre, we analysed short-, medium- and long-term outcomes in consecutive patients undergoing liver resection for colorectal liver metastases. A five-year retrospective review was carried out comparing the enhanced recovery programme to standard care.

**Results:**

A total of 172 patients were included in the analysis: 87 on standard care and 85 on an enhanced recovery programme. Open surgery was performed in 122 patients: 74 (85.1%) and 48 (56.5%) patients in the standard care and enhanced recovery programme, respectively (*p* < 0.001). There was a significant reduction in the median (IQR) length of hospital stay in the enhanced recovery programme compared with standard care (7 (5) days vs. 8 (3) days, *p* = 0.0009). There was no significant difference in survival between standard care and the Enhanced Recovery Programme at one (*p* = 0.818), three (*p* = 0.203), and five years (*p* = 0.247).

**Conclusion:**

An enhanced recovery programme was associated with a reduced length of hospital stay. There was no effect on the one-, three- and five-year survival.

**Supplementary Information:**

The online version contains supplementary material available at 10.1007/s00423-023-02968-4.

## Introduction

Enhanced recovery programmes (ERP) have demonstrated improved patient outcomes and length of stay following surgery by optimising the perioperative period [1]. Successful experiences with ERPs were reported as early as 1999 with the benefit of early and safe discharge of patients following major abdominal aneurysm surgery [2], and their use has since expanded into other specialities such as colorectal surgery [3]. It is a key peri-operative care recommendation by the National Institute for Clinical Excellence (NICE) [4] and is now the gold standard in many surgical specialties [5–9]. Through its successes, ERP has paved the way for contemporary prehabilitation strategies [10]. The use of ERPs following liver surgery began in 2008 following successful trials that showed earlier oral intake, a better postoperative period and a reduction in hospital stay [11]. A recent systematic review of patient outcomes following major liver surgery with ERP showed hospital stay to be reduced by 5–7 days without compromising morbidity and mortality [12]. Further, a recent randomised clinical trial comparing the ERP to standard care in open liver resection surgery showed a reduction in the incidence of medical complications and an improvement in quality of life [13]. The positive short-term effects of ERP have also been supported by other studies suggesting that such programmes are safe, cost-effective and acceptable to patients [14]

To date, although there have been reports on the effect of ERP on long-term survival of patients having surgery for colorectal cancer, there have been no reports on the effect of ERP on long-term survival in liver resection solely for colorectal liver metastases. The aim of this study was to clarify whether the well-established benefits of ERP may translate to reduced time to chemotherapy or increased likelihood of completing chemotherapy, thereby offering an overall survival benefit.

## Materials and methods

We evaluated retrospective data from a prospectively maintained database for patients who had undergone liver resection for colorectal liver metastasis (CRLM) between January 2011 and December 2016 at a regional hepatobiliary referral centre. Health Research Authority (HRA) guidelines on the use of NHS patient data for research purposes were adhered to [15]. All patients having liver resection for CRLM with curative intent were included regardless of the operative modality (laparoscopic or open). The ERP was introduced in January 2014 and patients were stratified into two groups based on time periods: Pre-ERP, hereafter referred to as control (CON) & ERP. We aimed to compare CON and ERP for an equivalent time period in years with a minimum 5-year follow-up. Patients were classified as ERP if they received at least 50% of the components in each of four domains (Appendix 1) of the ERP programme. CON patients received no components of ERP.

### Data collection

The data collated included patient demographics, details of surgical and oncological treatments, histology of resection specimens, duration of hospital stay, 90-day readmission, details of complications, compliance with individual ERP components and post-treatment survival (one-year, three-year and five-year mortality). The information was gathered from electronic records, clinic letters and operative notes. A multidisciplinary team of doctors, nurses, anaesthetists and allied health professionals contemporaneously completed an ERP document (Appendix 1). This document was developed through prior consultation with the multidisciplinary team (MDT). The primary outcome measured was posttreatment survival. One-, three- and five-year survival was scrutinised to determine whether there was any effect of ERP during this period. The secondary outcomes included hospital length of stay, post-operative complications using the Clavien-Dindo (CD) classification and 90-day readmission rates. The data was then reviewed and validated by all the investigators before being subjected to statistical analysis.

### Components of the ERP for liver surgery

ERP protocols involve a range of peri-operative strategies to promote recovery, facilitate safe and early discharge and improve patients’ overall peri-operative experience. All patients undergoing elective liver resection for CRLM were enrolled on to a standardised enhanced recovery programme from January 2014. The programme (Appendix 1) contained a series of strategies delivered by a specialist team as part of standard peri-operative care. The programme was assembled into four distinct but inter-related domains namely (i) Pre-operative Assessment & Information (ii) Day of Surgery Interventions (iii) Anaesthesia Protocols (iv) Post-operative Assessment, Information & Interventions. Each domain contained several components focussed on a particular aspect of patient optimisation, best clinical practice, and holistic patient care.

### Statistical analysis

Normality of data were assessed using Shapiro–Wilk tests. Univariate analyses (Mann–Whitney-*U* and chi^2^ tests) were used to detect significant differences in patient demographics and clinical case-mix between the CON and ERP cohorts (Table 1). Furthermore, patient demographics and clinical case mix features were analysed for significant associations with the one-, three- and five-year survival cohorts (Table 3). To achieve the primary aim of the study, survival analysis was applied to calculate one-, three- and five-year survival (postliver resection CON vs. ERP cohorts). Kaplan Meier survival curves were plotted and analysed using the log-rank test to determine significant differences in survival. All statistical analysis was performed using STATA statistical package version 15 (Stata Corp LLC4905 Lakeway Drive, College Station, Texas 77845-4512, USA) and figures were constructed in GraphPad Prism v 9.4.1 (GraphPad Software, San Diego, CA, USA). Significance was defined as *p* < 0.05.

**Table 1:** Patient demographics

Characteristic	Total	CON	ERP	*P*-value
Patient factors	172	87	85	
Mean age (years)	64.1	63.9	64.4	0.39
SD	10.1	9.4	10.8	
Male sex	125 (72.7%)	66 (75.9%)	59 (69.4%)	0.34
Mean BMI (kg/m^2^)	27.4	27.9	26.9	0.10
Risk stratification				
Mean Charlson Index	8.4	8.3	8.4	0.58
ASA I	12 (7.0%)	6 (6.9%)	6 (7.1%)	0.22
ASA II	94 (54.7%)	53 (60.9%)	41 (48.2%)	
ASA III	66 (38.4%)	28 (32.2%)	38 (44.7%)	
Surgical & oncological factors				
Open surgery	122 (70.9%)	74 (85.1%)	48 (56.5%)	< 0.001
Mean number of metastases	2.3	2.1	2.5	0.48
Mean number of liver segments	2.5	2.4	2.5	0.75
Major resection	94 (54.7%)	46 (52.9%)	48 (56.5%)	0.64
Neo-adjuvant chemotherapy	89 (51.7%)	44 50.6%	45 (52.9%)	0.76

*SD*, standard deviation; *BMI*, body mass index; *ASA*, American Society of Anaesthesiologist Classification; *ICU*, intensive care unit

## Results

**Fig. 1:**
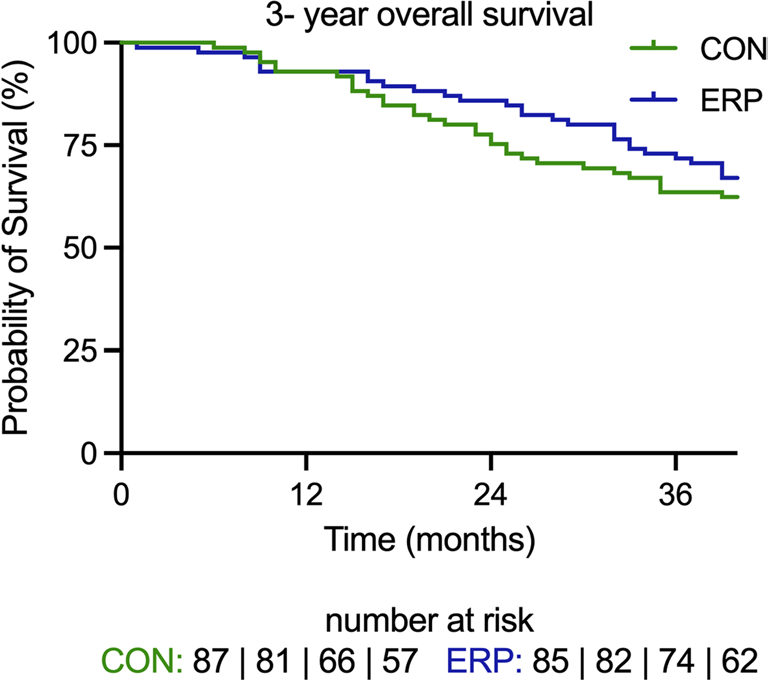
There was no effect of ERP on 3-year survival *p* = 0.203

**Fig. 2:**
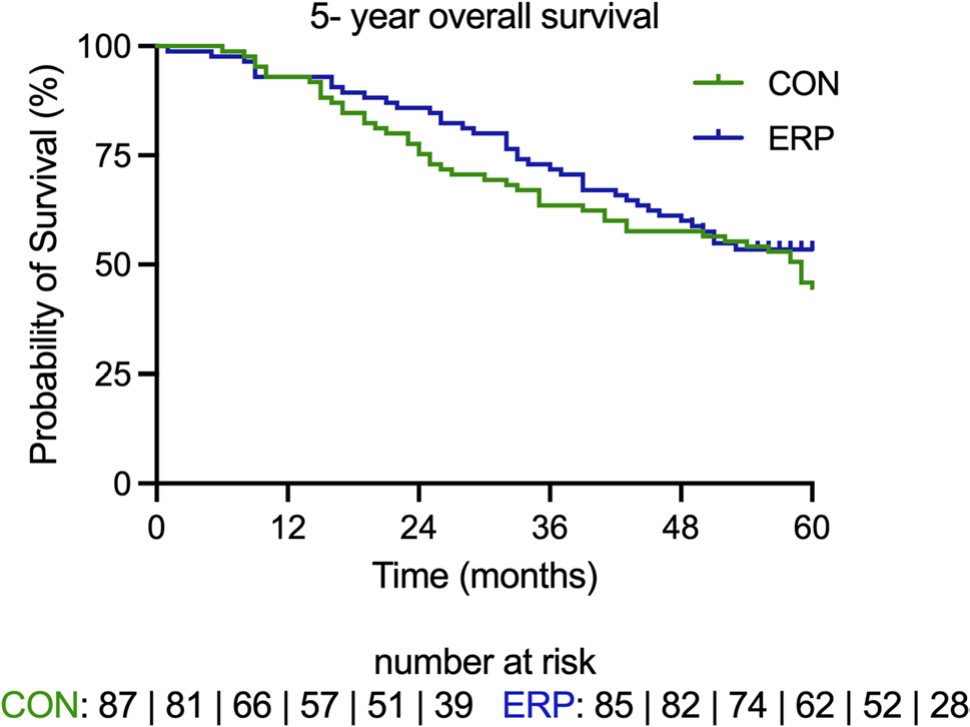
There was no effect of ERP on 5-year survival, *p* = 0.274

A total of 172 patients were included in the analysis, 87 CON and 85 ERP (Table 1). Sufficient data could not be retrieved for 19 patients (21.8%) in the CON and 10 patients (11.7%) in the ERP group. The median follow-up time was 58 months. One hundred and twenty-two patients had open surgery: 74 and 48 patients in the CON and ERP groups, respectively. Fifty patients had laparoscopic surgery: 13 and 37 in the CON and ERP groups, respectively. There was no significant difference in survival between CON and ERP at one, three and five years (Table 3). Although the median survival was greater in the ERP group compared to the CON group at three years, this was not significant (*p* > 0.05, Fig. 1). Furthermore, there was no significant difference in survival at five years (Fig. 2). We further assessed the determinants of survival (Table 3). The main determinant of 1-year survival was number of resected segments. The number of segments being inversely proportional to survival within the first year post-operatively. This effect was not observed at three and five years.

Over the five-year study period, the CON group had significantly more patients who underwent open surgery as compared to the ERP group; 74 (85.1%) vs. 48 (56.5%), *p* < 0.001. There was a significant reduction in the median (IQR) length of hospital stay in the ERP group as compared to the CON group (7 (5) vs. 8 (3) days, *p* = 0.0009). Open liver surgery was associated with a longer hospital stay compared with a laparoscopic approach (Table 2). Complications were grouped into minor (CD I-II) and major (CD III-IV). There was a statistical trend for differences in minor and major complications (data for CON vs. ERP, *p* = 0.088). However, re-admission rates between the groups were similar: CON 5 (0.05%) vs. ERP 9 (0.10%), *p* = 0.247 (Table 2). Interestingly, there were significantly more patients in the ERP group that experienced no complications; CON 33 (37.9%) vs. ERP 58 (68.2%), *p* < 0.001 (Table 3).

**Table 2:** Secondary outcomes

Length of hospital stay (days)	Total	CON	ERP	*P*-value
Median length of stay (IQR)	8.0 (3)	8.0 (3)	7.0 (5)	0.0009
Median ICU stay (IQR)	2.0 (2)	2.0 (2)	2.0 (2)	0.76
Complication rates				
No complications	91 (52.9%)	33 (37.9%)	58 (68.2%)	0.001
Clavien-Dindo I Clavien-Dindo II	33 (19.2%)	22 (25.3%)	11 (12.9%)	CD I-II vs. III-IV 0.08
	34 (19.8%)	26 (29.9%)	8 (9.4%)	
Clavien-Dindo III Clavien-Dindo IV	10 (5.8%)	4 (4.6%)	6 (7.1%)	
	2 (1.2%)	1 (1.2%)	1 (1.2%)	
Readmission rates	172	5 (0.05%)	9 (0.10%)	0.2471

*ICU*, intensive care unit

**Table 3:** Factors associated with overall survival in both CON & ERP groups

Variables	1-year survival	3-year survival	5-year survival
	*P*-value	*P*-value	*P*-value
Age (years)	0.90	0.40	0.10
Sex	0.72	0.90	0.86
BMI (kg/m^2^)	0.60	0.41	0.88
Charlson Index	0.42	0.11	0.18
Open surgery	0.43	0.80	0.50
Number of metastases	0.12	0.26	0.47
Number of segments	**0.02**	0.10	0.79
Neo-adjuvant chemotherapy	0.19	0.33	0.17
Length of stay (days)	0.70	0.08	0.06
Days in ICU	0.23	0.74	0.92
ASA	0.82	0.47	0.47

Bold indicates *p* < 0.05

Anaesthesiologist Classification; *ICU*, intensive care unit, *BMI*, body mass index; *ASA*, American Association of Anaesthesiology

## Discussion

Over the last decade, enhanced recovery programmes have become integrated as part of standard surgical perioperative practice [16]. Studies have cited a reduced length of hospital stay [17, 18], ICU stay [19], peri-operative complications [13] and improved patient experiences [20] as the main benefits of ERP. It is well estabished that the short-term benefits of improved functional recovery after major surgery translate into savings of bed-days and reduced cost to healthcare systems [21, 22]. Our study is consistent with the literature regarding length of hospital stay. This association was recently corroborated by Noba et al., in their meta-analysis of 3739 patients undergoing liver resections [23].

In elective cancer surgery, the effect of ERP on improving short- and medium-term outcomes such as functional recovery and hospital stay has been well established [16]. However, there is a paucity of literature on the effect of ERP on long-term outcomes such as survival. Curtis et al. assessed the effect of ERP on 5-year survival in a colorectal cancer cohort of 854 patients and demonstrated improved survival when combined with a laparoscopic approach [24]. To our knowledge, this is the first study that has attempted to assess the effect of ERP on medium and long-term survival in curative liver resection for CRLM. In our series, ERP was found to be associated with a significant reduction in the median length of hospital stay. This finding was consistent with other studies that have analysed ERP in the context of elective hepatopancreatobiliary [5, 25], colorectal [6, 26, 27], oesophagogastric [7, 28] urological [8, 29] and breast surgery [9]. Although length of stay may be influenced by other administrative and social factors outside of ERP [30], most investigators believe this observation to be weighted in favour of ERP.

We found no significant relationship between the ERP and survival at one, three, or five years. This was not an unexpected finding, considering that ERP interventions are more likely to influence homeostatic and physiological factors than the biology of the disease [31]. More importantly, within a cancer context, it may also be that ERP has limited influence on reducing time to adjuvant chemotherapy or chances of completion. In one colorectal cancer study, ERP was associated with ‘on time’ initiation of adjuvant chemotherapy; however, long-term survival was not studied [32]. We would expect that interventions that lead to quicker functional recovery may offer an increased opportunity for patients to shorten their time to commencing adjuvant therapy. Some studies have alluded to this effect and have shown improved survival as a function of a reduced time to completion of adjuvant chemo or radiotherapy [33, 34]. This effect was not investigated in our study and provides an avenue for further analysis.

We analysed several factors to determine those common to both groups that may have influenced survival. We found that independent of ERP, the number of resected segments had a significant association with survival at one year. A greater number of resected segments was associated with poorer survival at one year. This finding was not dissimilar to work done by Fromer et al. who demonstrated poorer survival with > 3 resected lesions [35]. Evidence has suggested that the number of metastases may be directly related to tumour burden. The number of metastases and their their anatomical distribution (uni/bi lobar) may serve as a marker for overall disease burden and may suggest more aggressive tumour biology, hence poorer survival [36].

Concerning length of stay, the ERP group showed a statistically significant reduction when compared to the CON group. The clinical importance of a reduction in LoS by a day must be viewed within the wider context of patient satisfaction and the cost reduction in total number of bed days. This was beyond the scope of this study. It is possible that this observation could be related to smaller numbers of patients in the CON group (13 patients) who also had laparoscopic surgery. Reduced length of stay in ERP has been extensively investigated by several authors. While authors suggest that hospital stay may be influenced by other factors such as social care provision, administrative protocols and patient-related factors [30], there appears to be a genuine effect of accelerated hospital recovery with ERP that seems to be consistent with a wide range of ERP protocols [16].

In our series, there was no difference in the incidence of major complications (CD III-IV) between the groups. However, significantly more patients in the ERP group had no complications when compared with the CON group. While this could be a genuine effect, it is also plausible that this observation could be explained by the higher proportion of patients having open surgery in the CON group and having complications related to this. Contrary to this assumption, a study by Jones et al. found ERP to be associated with reduced complication rates when investigating a cohort of patients undergoing open liver resections [13]. Another series of primary liver resections found reduced complication rates with ERP; however, this was confined to patients with the highest compliance [37]. Additionally, a recent meta-analysis of 27 comparative liver resection studies showed a significant reduction in both length of stay and complication rates [23]. It is our opinion that while ERP may have no influence on surgical factors (such as technical failures) that may lead to complications; peri-operative optimisation with better glycaemic control and anaemia correction and maintaining intra-operative haemostasis along with postoperative strategies may reduce the risk of complications.

ERPs have evolved in step with other improvements in perioperative care such as multi-modal analgesia, patient-tailored anaesthesia and more widespread use of objective operative risk stratification tools such as cardiopulmonary exercise testing (CPET) [38]. In light of these improvements over the last decade and the multi-modal nature of ERP, delineating what may be the most important components of these pathways can be challenging due to wide variations in adherence rates and number ERP components [39]. It is widely accepted that observed improvements may be due to the collective implementation of several components and that the effectiveness of ERPs are enshrined in the multimodal nature of their delivery [31].

## Strengths and limitations

This study is the first of its kind to address the impact of ERP on medium and long-term survival for the curative resection of CRLM. Due to the wide variability in content and implementation of ERPs, coupled with demographic differences and care pathways across NHS Trusts, we concluded that a single site observational study was most appropriate in addressing this issue. Data was collected using robust reporting and recording systems and was verified for accuracy by all authors. Our findings supplement the existing literature on ERP and provides evidential basis for further larger controlled studies powered to detect improvements in long-term survival. We believe this to be a pertinent area for further investigation due to the evolving nature of ERP and the more widespread uptake of minimally invasive stragegies such as laparoscopic and robotic surgery [40].

Inherent biases within our study design meant that findings should be interpreted within the limitations of a retrospective study. The nature of ERP and the advent of its implementation at our institution may have been associated with recall and reporting biases that may have over-estimated the beneficial effects of ERP. We concede that within the period that ERP commenced, data may have been more fastidiously recorded and reported especially within the context of being a nationally recognised quality indicator of peri-operative patient care. Additionally, the ERP programme coincided with an increased uptake of laparoscopic liver resection. We are aware that minimally invasive techniques are associated with reduced complication rates and length of hospital stay in some series [41]. We recognise that inadequate numbers of study patients are likely to preclude revealing significant differences in low incidence secondary outcomes. In most surgical units within the National Health Service, ERP is now standard practice[42]. Further studies within this field may provide more clarity on medium and long-term survival by probing larger databases. As the components within ERP are improved and evolve further, so too will oncological and surgical techniques. Improving survival in patients with CRLM may depend on a range of strategies deployed via a multimodal, multidisciplinary platform.

## Conclusion

An enhanced recovery programme accelerates recovery and reduces hospital stay in patients undergoing curative resection for CRLM. We found ERP to have no effect on long-term survival .

### Supplementary Information

Below is the link to the electronic supplementary material.

Supplementary file1 (PDF 677 KB)

## Data Availability

The data are available from the corresponding author upon reasonable request.
