# Supplementary material for: The effect of the enhanced recovery programme on long-term survival following liver resection for colorectal liver metastases
Source: Langenbecks Arch Surg. 2023 Jun 19;408(1):239. doi: 10.1007/s00423-023-02968-4 (PMC10279559; doi:10.1007/s00423-023-02968-4)
Supplement: Supplementary file 1 — Supplementary file1 (PDF 677 KB) [file 423_2023_2968_MOESM1_ESM.pdf]

APPENDIX 1

PROOF

Elective Liver Resection Enhanced Recovery Protocol

NHS
East Lancashire Hospitals
NHS Trust
A University Teaching Trust

(Attach patient label here)

Hospital No:................................ DOB:................................

First Name:................................ M / F:......... Religion:................................

Last Name:................................ GP:................................

Address:..

....

NHS No:..

Admission Date: ____________________

Expected Length of Stay: ____________________

Planned Discharge Date: ____________________

Consultant: ____________________

This protocol is to be used as a prompt for compliance with the Enhanced Recovery patient pathway. It does not constitute a prescription. Please ensure the document is commenced in Pre-operative Assessment and accompanies the patient at all times and is utilised during nursing handovers and ward rounds.

Please ✓ for all instructions achieved and ✗ if not achieved and document as a variance with rationale. If any instruction is inappropriate for an individual patient then that should be altered and documented as a variance with rationale. The patient will still benefit from the remainder of the protocol.

Day 0 Patient admission
Day 4-5 Expected discharge date for laparoscopic liver resection
Day 8-10 Expected discharge date for open/laparascopic converted procedure

Date	Staff Name	Signature	Initial	Designation	Bleep/ Ward

PROOF

Enhanced Recovery Programme (ERP) Guidelines for Surgery

Each operation will have a protocol that is specific to that surgery. Please use these to guide you. Any instructions not achieved should be documented as a variance with rationale in the ward documentation/notes

Please see the ELHT intranet (under anaesthetics) for ERP guidelines on:-

- Enhanced Recovery Operational Policy
- Perioperative Fasting guideline
- Anaesthesia guidelines for Liver
- Anaesthesia Enhanced Recovery guidelines
- Goal Directed Fluid Therapy (GDFT) guidelines
- Postoperative Hypotension / Epidural Management guideline on the Enhanced Recovery Programme
- Fluid guidelines (ELHT IV Fluid therapy guideline - Adults)
- Acute Pain Team Guidelines
- Mobilising Enhanced Recovery Patients on Critical Care guideline

Pre-Operative Assessment **Date:** ______________________________

PROOF

**Protocol Instruction**	**Achieved √ or x**	**Signature**
Verbal information on Enhanced Recovery given		
Enhanced Recovery booklet given and explained		
Eido leaflet given on operation		
Eido leaflet given on anaesthetic		
Eido leaflet given on pain relief after surgery		
Information leaflet on venous thromboembolism (VTE) given		
Preventing falls in hospital information leaflet given		
Smoking cessation, health and wellbeing advice given		
Deep breathing and limb exercises advice given		
Importance of post-operative nutrition advice given		
Importance of post-operative early mobilisation advice given		
Preload sachets given with printed information sheet (unless a diabetic on insulin)		
Patient advised to bring sugar free chewing gum		

PROOF

Day before Surgery **Date: ______________________________**

Protocol Instruction	Achieved √ or x	Signature
Normal diet		
If INR ≤1.5 give low molecular weight Heparin at 18:00 hours (to be given in leg)		

Day of Surgery Pre-Operatively **Date: ______________________________**

Weigh patient on admission and document (Kgs) in notes		
Clear fluids only from midnight		
Preload at 06.00		
Nil by mouth from 06:00 after Pre-load		
Pre-operative Gabapentin given		
Antiembolic stockings in situ		
Post-operative pain relief advice given		
Deep breathing and limb exercises advice reiterated		
Importance of post-operative nutrition advice reiterated		
Importance of early mobilisation advice reiterated		
Check patient has ERP booklet and advise to complete post-operatively		

PROOF

This page is to be completed in theatre by theatre staff

Anaesthesia:

Protocol Instruction	Achieved √ or x	Signature
Intra-operative Goal Directed Fluid Therapy (GDFT)		
GDFT documentation completed		
Primary analgesic technique as per Acute Pain Team guideline		
Multimodal antiemesis intraoperatively		
Maintain normothermia		
Post-op fluid prescribed as per ELHT IV Fluid therapy guidelines - Adult		
Referral form to Acute Pain Team completed		
Post-operative primary and rescue analgesia prescribed		
Post-operative Paracetamol prescribed regularly		
Post-operative Gabapentin prescribed regularly		
Post-operative Buccastem prescribed regularly		

OPCS Coding:

Liaise with the surgeon and ensure the correct 5-digit OPCS codes for surgery and co-morbidities have been entered into TheatreMan post-operatively		
For Intra-operative goal directed fluid therapy (GDFT) please ensure the OPCS code Y73.6 has been added		

Day of Surgery Post-Op (Day 0) **Date:** ____________________________ **Ward:** __________________

PROOF

Communication

Protocol Instruction	Achieved √ or x	Signature
Follow post-operative instructions		
Patient aware of today's goals		
Encourage patient to complete their diary		

Pain

Monitor pain and side-effects of analgesia. Please use the primary analgesic observation sheet and Acute Pain Team guidelines for advice		

Deep Breathing & Mobility

All staff to promote deep breathing exercises		
Sit patient upright in bed within 6 hours of surgery		
Antiembolic stockings in situ		
Flotrons in situ		
All staff to promote leg exercises		

Eating & Drinking

Ensure Buccastem is prescribed and given regularly		
Encourage free fluids immediately		
Fluid input IV and or oral as per Trust guidelines.		
Ensure any temporarily unused cannulas are flushed PRN to remain patent. Complete Visual Infusion Phlebitis (VIP) score		
Encourage three supplement drinks a day		
Offer evening meal / light diet		

Elimination

Maintain accurate fluid balance		
Document bowel sounds and movements		

Infection

Maintain normothermia, report pyrexia to surgeons		
Check surgical wound, dressing, drains		

Observations

Early Warning Score (EWS) documented with regular observations		

PROOF

Day 1 Post-Op **Date:** ______________________________ **Ward:** ___________________

Communication

Protocol Instruction	Achieved √ or x	Signature
Patient aware of today's goals		
Encourage patient to complete their diary		

Pain

Ensure Acute Pain Team aware of patient		
Follow Acute Pain Team plan		

Deep Breathing & Mobility

All staff to promote deep breathing exercises		
All staff to promote leg exercises		
Referral to Physiotherapy		
If INR ≤1.5 give low molecular weight Heparin at 18:00 hours (to be given in leg)		
If INR ≥1.5 let surgeons know		
Antiembolic stockings in situ		
Sit patient out of bed in chair: (If in Critical Care, see mobilising ERP patients guideline) — For breakfast		
For lunch		
For evening meal		
Walk 60 metres (once)		
Walk 60 metres (twice)		

Eating & Drinking

Referral to dietitian if MUST score ≥2		
Buccastem given regularly		
Encourage free fluids		
Fluid input IV and or oral as per Trust guidelines.		
Ensure any temporarily unused cannulas are flushed PRN to remain patent. Complete Visual Infusion Phlebitis (VIP) score		
Gives 3 supplement drinks a day, chart on fluid balance: — Drink 1		
Drink 2		
Drink 3		
Encourage three meals a day in addition to supplements		
Sugar free chewing gum given 15 mins tds		

Elimination

Maintain accurate fluid balance		
Document bowel sounds and movements		

Infection

Report pyrexia to surgeons		
Surgical review, consider drain removal		
Check surgical wound, dressings		

Observations

Early Warning Score (EWS) documented with regular observations		

PROOF

Day 2 Post-Op **Date:** ______________________________ **Ward:** ___________________

Communication

Protocol Instruction	Achieved √ or x	Signature
Patient aware of today's goals		
Encourage patient to complete their diary		
Begin discharge planning, see discharge plan on back page		

Pain

Ensure Acute Pain Team aware of patient		
Follow Acute Pain Team plan		

Deep breathing & Mobility

All staff promote deep breathing exercises			
All staff to promote leg exercises			
If INR ≤1.5 give low molecular weight Heparin at 18:00 hours (to be given in leg)			
If INR ≥1.5 let surgeons know			
Antiembolic stockings in situ			
Sit patient out of bed in chair: (If in critical care, see mobilising ERP patients guideline)	For breakfast		
	For lunch		
	For evening meal		
Encourage mobilising to dining room or day room where available			
Aim for 4 x 60 metres walks a day:	Walk 1		
	Walk 2		
	Walk 3		
	Walk 4		

Eating & Drinking

Buccastem given regularly			
Encourage free fluids			
Fluid input IV and or oral as per Trust guidelines.			
Ensure any temporarily unused cannulas are flushed PRN to remain patent. Complete Visual Infusion Phlebitis (VIP) score			
Gives 3 supplement drinks a day, chart on fluid balance:	Drink 1		
	Drink 2		
	Drink 3		
Encourage three meals a day in addition to supplements			
Sugar free chewing gum given 15 mins tds			
Weigh patient and document weight (kg) in notes			

Elimination

Maintain accurate fluid balance		
Document bowel sounds and movements		

Infection

Maintain normothermia, report pyrexia to surgeons		
Surgical review, consider drain removal, urinary catheter removal		
Check surgical wound, dressings		

Observations

Early Warning Score (EWS) documented with regular observations		

Day 3 Post-Op **Date:** ______________________________ **Ward:** ___________________

PROOF

Communication

Protocol Instruction	Achieved √ or x	Signature
Patient aware of today's goals		
Encourage patient to complete their diary		
Review discharge planning, see discharge plan on back page		

Pain

Ensure Acute Pain Team aware of patient		
Follow Acute Pain Team plan / see weaning plan		

Deep Breathing & Mobility

All staff to promote deep breathing			
All staff to promote leg exercises			
If INR ≤1.5 give low molecular weight Heparin at 18:00 hours (to be given in leg)			
If INR ≥1.5 let surgeons know			
Antiembolic stockings in situ			
Sit patient out of bed in chair:	For breakfast		
	For lunch		
	For evening meal		
Encourage mobilising to dining room or day room where available			
Aim for 4 x 60 metres walks a day:	Walk 1		
	Walk 2		
	Walk 3		
	Walk 4		

Eating & Drinking

Buccastem given regularly			
Encourage free fluids			
Fluid input IV and or oral as per Trust guidelines.			
Ensure any temporarily unused cannulas are flushed PRN to remain patent. Complete Visual Infusion Phlebitis (VIP) score			
Gives 3 supplement drinks a day, chart on fluid balance:	Drink 1		
	Drink 2		
	Drink 3		
Encourage three meals a day in addition to supplements			
Sugar free chewing gum given 15 mins tds			

Elimination

Maintain accurate fluid balance		
Document bowel sounds and movements		

Infection

Maintain normothermia, report pyrexia to surgeons		
Surgical review, consider drain removal, urinary catheter removal		
Check surgical wound, dressings		

Observations

Early Warning Score (EWS) documented with regular observations		

PROOF

Day 4 Post-Op **Date:** ______________________________ **Ward:** ____________________

Communication

Protocol Instruction	Achieved √ or x	Signature
Patient aware of today's goals		
Encourage patient to complete their diary		
Review discharge planning, see discharge plan on back page		

Pain

Ensure Acute Pain Team aware of patient		
Follow Acute Pain Team plan / see weaning plan		

Deep Breathing & Mobility

All staff to promote deep breathing		
All staff to promote leg exercises		
If INR ≤1.5 give low molecular weight Heparin at 18:00 hours (to be given in leg)		
If INR ≥1.5 let surgeons know		
Antiembolic stockings in situ		
Sit patient out of bed in chair: For breakfast		
For lunch		
For evening meal		
Encourage mobilising to dining room or day room where available		
Aim for 4 x 60 metres walks a day: Walk 1		
Walk 2		
Walk 3		
Walk 4		

Eating & Drinking

Buccastem given regularly		
Encourage free fluids		
Fluid input IV and or oral as per Trust guidelines.		
Ensure any temporarily unused cannulas are flushed PRN to remain patent. Complete Visual Infusion Phlebitis (VIP) score		
Gives 3 supplement drinks a day, chart on fluid balance: Drink 1		
Drink 2		
Drink 3		
Encourage three meals a day in addition to supplements		
Sugar free chewing gum given 15 mins tds		
Weigh patient and document weight (kg) in notes		

Elimination

Maintain accurate fluid balance		
Document bowel sounds and movements		

Infection

Maintain normothermia, report pyrexia to surgeons		
Surgical review, consider drain removal, urinary catheter removal		
Check surgical wound, dressings		

Observations

Early Warning Score (EWS) documented with regular observations		

Day 5 and Onwards PROOF **Date:** ______________________________ **Ward:** ___________________

Communication

Protocol Instruction	Achieved √ or x	Signature
Patient aware of today's goals		
Encourage patient to complete their diary		
Review discharge planning, see discharge plan on back page		

Pain

Ensure Acute Pain Team aware of patient		
Follow Acute Pain Team plan / see weaning plan		

Deep Breathing & Mobility

All staff to promote deep breathing		
All staff to promote leg exercises		
If INR ≤1.5 give low molecular weight Heparin at 18:00 hours (to be given in leg)		
If INR ≥1.5 let surgeons know		
Antiembolic stockings in situ		
Sit patient out of bed in chair: For breakfast		
For lunch		
For evening meal		
Encourage mobilising to dining room or day room where available		
Aim for 4 x 60 metres walks a day: Walk 1		
Walk 2		
Walk 3		
Walk 4		

Eating & Drinking

Buccastem given regularly		
Encourage free fluids		
Fluid input IV and or oral as per Trust guidelines.		
Ensure any temporarily unused cannulas are flushed PRN to remain patent. Complete Visual Infusion Phlebitis (VIP) score		
Gives 3 supplement drinks a day, chart on fluid balance: Drink 1		
Drink 2		
Drink 3		
Encourage three meals a day in addition to supplements		
Sugar free chewing gum given 15 mins tds		

Elimination

Maintain accurate fluid balance		
Document bowel sounds and movements		

Infection

Maintain normothermia, report pyrexia to surgeons		
Surgical review, consider drain removal, urinary catheter removal		
Check surgical wound, dressings		

Observations

Early Warning Score (EWS) documented with regular observations		

For additional days, please continue to document any protocol instructions not achieved as a variance in the notes.

PROOF

Discharge Planning:

Referrals to consider:

Protocol Instruction	Date	Initial
Referral to Carers Service to support relatives caring for the patient upon discharge		
Referral to social worker if needed		
Referral to occupational therapist if needed		

Instruction	Date	Initial
Drugs ordered to take home		
Patient / family competent to administer subcutaneous low molecular weight heparin.<br>If not District Nurse organised		
District Nurse referral made if required		
Date for removal of clips by practice nurse		
Outpatient appointment made		
Ward telephone number given		
Transport if needed		
Post op care advice given		
Complete discharge checklist on Elective Surgical Pathway (Blue) on day of discharge		

Date: ______________________ Time: __________________________ Surgically fit for discharge

Date: ______________________ Time: __________________________ Of actual discharge
